# The influence of climatic variation and density on the survival of an insular passerine *Zosterops lateralis*

**DOI:** 10.1371/journal.pone.0176360

**Published:** 2017-04-28

**Authors:** Erik M. Sandvig, Tim Coulson, Jiro Kikkawa, Sonya M. Clegg

**Affiliations:** 1 Department of Zoology, University of Oxford, Oxford, United Kingdom; 2 Edward Grey Institute for Field Ornithology, Department of Zoology, University of Oxford, Oxford, United Kingdom; 3 School of Integrative Biology, University of Queensland, Brisbane, Australia; Universita degli Studi di Milano-Bicocca, ITALY

## Abstract

Understanding the influence of environmental factors on population dynamics is fundamental to many areas in biology. Survival is a key factor of population biology, as it is thought to be the predominant driver of growth in long-lived passerines, which can be influenced by both biotic and abiotic environmental conditions. We used mark-recapture methods and generalized linear mixed models to test the influence of density and climatic variation, measured at a regional and local scale (Southern Oscillation Index [SOI] and rainfall, respectively), on seasonal variation in survival rates of an insular population of Silvereyes (*Zosterops lateralis chlorocephalus*), during a 15-year study period, off the east coast of Australia. We found overall high survival rates for adults and juveniles (81% and 59%, respectively). Local scale climate (i.e. rainfall) and density were the principal environmental factors influencing their survival, both with a negative relationship. A significant interactive effect of density and rainfall influenced survival as they both increased. However, survival remained low when density was at it highest, independent of the amount of rainfall. Nestling survival was negatively influenced by rainfall and density, positively by SOI, and chicks that hatched later in the breeding season had higher survival rates. The regional scale climate variable (i.e. SOI) did not explain survival rates as strongly as rainfall in any age class. Our results contribute to the understanding of insular avian population dynamics and the differential effects of environmental factors across age classes. Climatic predictions expect El Niño events to increase, meaning dryer conditions in eastern Australia, potentially increasing Silvereye survival across age classes. However, the long-term effect of lower rainfall on food availability is unknown; consequently, the outcome of lower rainfall on Silvereye survival rates is uncertain.

## Introduction

Understanding how climatic variation and density-dependent effects influence population dynamics is a topic of great interest in ecology, particularly as environmental change is occurring at an unprecedented rate [1]. Climatic fluctuations can impact populations directly through physiological processes [2], as well as through interaction with intrinsic density-dependent processes [3]. In birds they have been linked to changes in distribution, phenology, behaviour, morphology and population dynamics [4–7]. In avian population dynamics, of the main components that govern variation in population growth (survival, fecundity, immigration and emigration), adult survival rate is thought to be the predominant driver of change in growth in long-lived species [8]. Research has shown that large-scale climate patterns, like El Niño/Southern Oscillation (ENSO), play an important role in bird population dynamics [9], driving sharp increases and steep declines in population sizes [10–12], as well as impacting phenology and breeding success [13,14]. Thus, to improve our understanding of how climatic variation influences avian population dynamics it is key to understand the links between climate, density-dependence and survival.

ENSO influences climate on a global scale [15], driving temperature and rainfall patterns measured at a local scale [16]. Survival in birds is closely linked to changes in temperature and rainfall during El Niño events [17,18]. Furthermore, rainfall may be the most important short-term factor influencing the demography of tropical and subtropical birds, due to increased food availability, linked directly or indirectly to rainfall [19]. However, in events of extreme precipitation it may decrease breeding success and increase mortality [20]. These stochastic environmental fluctuations have been found to interact with density-dependent effects and influence bird demographics, decreasing survival when climatic conditions are harsh and density is high in temperate species [21], however much less is known about these interactions for subtropical species [22].

Studies of the effects of ENSO on insular birds are mostly restricted to seabirds, and to influences on demography (survival and breeding success) [5,14,23,24]; less attention has been given to the impacts on insular terrestrial birds. The avifauna of the Galapágos provide the best exemplars of the impacts of ENSO events on insular terrestrial passerines, where it has been linked to breeding success and even rapid phenotypic change [10,11,13]. On arid islands, such as the Galapágos, ENSO events and specifically rainfall trigger dramatic increases in population size of passerine species [13].

Indices of large-scale climate patterns, such as ENSO and NAO (North Atlantic Oscillation), are commonly used to assess the impact of climate variation on population dynamics [25–27] and in some cases are argued to be better predictors of demographic rates than local scale climate measures [28]. However, these indices simplify a sum of climate components (e.g. rainfall, temperature, winds) acting over very large geographical areas, where in their place local climate measures may be better predictors of local demographics. Thus, it is important to identify which climate variable is the most appropriate.

In this study, we evaluate the influence of climatic factors (ENSO and rainfall) and population density on the survival of an insular population of a silvereye subspecies (*Zosterops lateralis chlorocephalus*). We used mark-recapture techniques to analyze a 15-year dataset of a small, closed population, restricted to Heron Island, Australia. We assess the influence of climatic variation using variables summarizing climate at both broad and local geographic scales, as well as the influence of population density on the survival probabilities among seasons across three silvereye age classes. This study will contribute to the understanding of the impacts of environmental factors during different stages of the life cycle of insular birds in the less studied subtropical areas.

## Methods

### Study site and species

Heron Island (23° 26’ S, 151° 57’ E) is a wooded island of the Capricorn-Bunker Group, covering an area of approximately 17 ha, located 70km from the eastern Australian Coast on the southern Great Barrier Reef. Mean annual rainfall is 1027.8 mm (1956–2007), and mean max and min temperatures are 26.2°C and 20.8 ^*^C (1962–2007), respectively (Australian Bureau of Meteorology, http://www.bom.gov.au). The Capricorn silvereye (*Zosterops lateralis chlorocephalus*) is the only regularly breeding passerine on the island [29]; it is a long-lived species, reaching ages of up to 16 years; juveniles reach breeding age in the summer the year after hatching [30]. Individuals within the population have been color-banded since 1964 and survival and fecundity were monitored in detail from 1979 to 1993 [31], under the ethics approval from the University of Queensland. The breeding population size varies between 250 and 500 individuals [32]. Active searches for nests were conducted throughout the island during the breeding season and nests were subsequently monitored until the last nestling fledged. The number of eggs that hatched in each nest was counted, as well as the number of nestlings that fledged [33]. Description of the species and details of field methods during the study period are given in [30,33,34]. Between 1980 and 1993 the percentage of un-banded birds in the population ranged between 0.3% and 4.2% (one to 16 individuals)[32], while movement among the islands of the Capricorn-Bunker Island Group is estimated to be <1% per generation, making the population effectively closed [33].

### Capture histories

Capture-recapture (CR) history data were obtained from individually color-banded birds captured and resighted (recaptured) between 1979 and 1993, constituting a dataset of 60 time-steps. Each time step represents a season, corresponding to spring (September), summer (October-January), autumn (February-March) and winter (May-July). Although bird censuses were usually made during the same months each year, frequency of visits meant that on some occasions sampling months varied between years: birds were censused in all four seasons most years, but no resighting censuses were conducted during spring 1979, spring 1980, autumn 1981, autumn 1982 and winter 1993. Most birds were first captured as nestlings, and the remainder as juveniles and adults. All nestlings were first captured during summer; juveniles in summer, autumn or winter; and the remaining birds as adults throughout the year. Because nestlings transition to juveniles within the summer season (nestlings fledge at 10–12 days)[34], they violate the CR model assumption that individuals transition age classes between time-steps, not within them [35]. Therefore, nestling survival was analyzed using a generalized linear model approach (n = 6194), described below.

A capture history dataset was created for all nestlings that survived to juvenile stage, and individuals first captured as juveniles or adults (n = 4345). The first capture occasion of nestlings was suppressed in the capture history and was reclassified to the new age class according to the season in which they were first resighted (i.e. summer, autumn and winter of hatch year were reclassified as juvenile; next spring and onwards as adult). Birds that were first banded as nestlings, but that were not resighted as independent birds were removed from the capture history dataset (n = 2530)—those individuals were still considered in the nestling models. All juveniles were considered to transition to the adult age in their first spring after hatching; they remained as adults until death. The number of juveniles born and captured during their first-year spring was too low (n = 39) to test this season for juveniles as a variable in the models. Thus, only summer, autumn and winter were included for juveniles.

### Environmental covariates

To test the effects of environmental variation on apparent survival we selected a broad scale climate covariate, the Southern Oscillation Index (SOI) and rainfall as a local scale weather covariate. The SOI measures the oscillation in surface air pressure between the tropical eastern and western Pacific Ocean waters and influences broad scale weather patterns in the Pacific [26]. The SOI has been shown to influence population dynamics of a wide range of species [10,36–41]. Rainfall recorded on the island was used as the local scale weather covariate. For silvereyes on Heron Island, moderate rainfall has been associated with good food supply, but causes nest failure when high [42], as well as high adult and first year mortality in extreme weather events, like cyclones, when intense rainfall is accompanied by strong winds [32,43]. The effect of cyclones was not included in the analysis due to a low recurrence of these events, where only two cyclones impacted the island during the study period, from which the population recovered rapidly in both occasions [32]. For nestlings, we used the monthly SOI value of the month in which the nestling hatched, while for rainfall we calculated the mean rainfall over the 12-day nestling period (Rain12) for each nest, starting from hatch day. To test for the importance of rainfall earlier and later in the nest occupancy period, we also calculated mean rainfall for the first and last six days (RainF6 and RainL6, respectively). For juveniles and adults, monthly SOI values were averaged over three-month census periods. Daily rainfall values were averaged over three-month census periods, from now on referred to as seasonal rainfall (SR). To assess if SR has a lagged effect on food supply and hence survival, we added a one-month lag to the three-month rainfall averages, as well as a one-month lagged SOI effect. SOI and rainfall data were obtained from the Australian Bureau of Meteorology (www.bom.gov.au). We were unable to include temperature as a covariate in the analysis due to a large gap in the dataset of the Australian Bureau of Meteorology that completely overlapped with our study period. To test for an effect of the time of the breeding season in which nestling hatched, we assigned a number to each nest from the earliest nest hatch date to the latest according to the breeding season in which that nest was found.

### Density

Density was calculated as the total number of juveniles and adults resighted during each season. Nestlings were not included because we assumed they were not strong competitors, being dependent upon their parents, and would not influence survival or recapture rates of independent birds.

### Nestling survival analysis

The nestling period is temporally different to juvenile and adult age classes and was thus analyzed separately. We constructed generalized mixed linear models (GLMMs) in the package lme4 [44] with a binomial error structure in R (R Core Team 2015), with one of two binomial response variables–the number of nestlings that survived to day 8 when they were banded (n = 4122) versus number that hatched (n = 6194), and number of nestlings that fledged (n = 3922) versus number that hatched (n = 6194). We included combinations of 10 fixed effects in each model: mean 12-day rainfall (Rain12), mean rainfall of first six nest days (RainF6), mean rainfall of last six nest days (RainL6), SOI, hatch date, quadratic effect of Rain12, quadratic effects of RainF6 and RainL6, quadratic effect of SOI and population density. Two random effects were also included, year, and pair identity (Pair ID), since the same pair could have multiple clutches in one breeding season. Model selection was conducted by first building two alternate full models and then excluding variables one at a time. Model 1 included all variables except for RainF6 and RainL6; the other included all variables except for Rain12 (Model 2). Thus, the two models were as follows:

$$\text{Probability}\;\text{of}\;\text{Survival}∼\text{Rain}12+\text{Rain}12^{2}+\text{SOI}+{\text{SOI}}^{2}+\text{Hatch}\;\text{Date}+\text{Density}+(1|\text{Year})+(1|\text{Pair}\;\text{ID})$$ Model 1

$$\text{Probability}\;\text{of}\;\text{Survival}∼\text{RainF}6+\text{RainF}6^{2}+\text{RainL}6+\text{RainL}6^{2}+\text{SOI}+{\text{SOI}}^{2}+\text{Hatch}\;\text{Date}+\text{Density}+(1|\text{Year})+(1|\text{Pair}\;\text{ID})$$ Model 2

Models were ranked using Akaike’s Information Criterion (AIC), considering a ΔAIC > than two, to identify the model of best fit [45].

### Juvenile and adult survival analysis

For juvenile and adult CR data, goodness of fit tests (GOF) were performed in the program U-CARE [46]. We found a lack of fit due to a “transience” effect in juveniles (1.798, p = 0.036), and in adults (8.351, p = 5.551e-017). Due to the closed nature of our study system, transient individuals are extremely unlikely; and the effect we detected was most likely due to juvenile transition to the adult age class, rather than the movement of juveniles off the island. Thus, we account for the transition between juvenile and adult age classes by maintaining an age class effect in every model. The trap dependence test showed a significant “trap-happiness” effect (juveniles: -3.692, p = 0.0002; adults: -9.527, p = 0), where trapped individuals are more likely to be trapped again than un-trapped individuals. We accounted for this by incorporating a trap-awareness transition matrix to the model, which treats individuals as having a trap response immediately after being trapped but reverting to a naïve state if not recaptured the next trapping session [47] (S1A, S1B and S1C Table).

We developed a single-state CR model, where individuals were observed or not observed at each time step. We analyzed 4,345 individual CR histories to estimate survival probabilities across seasons using standard CR statistical analysis [35] implemented in the program E-SURGE [48]. CR methods explicitly take into account the probability of an individual being alive but not recaptured by modeling survival and recapture probabilities simultaneously [35]. They are also flexible, allowing assessment of covariates on the variation of recapture and survival rates over time. All the environmental covariates and density values were rescaled to have a mean of zero and standard deviation of one.

Model selection was conducted by first testing if survival and detection rates were constant or varied among seasons. Because the recapture rate was below one and varied between years, we investigated which recapture model best fit our data. Recapture models considered yearly, seasonal and age variation, as well as additive effects of density, SOI and SR. We then built survival models accounting for yearly, season and age variation, as well as linear and quadratic effects of the environmental covariates, and tested them simultaneously with our optimal recapture model. The time to achieve model fit was considerable due to the number of parameters being estimated for variation between every time-step and age; hence we grouped time-steps into seasons across all years. From our base model of season by age we then approached model selection by considering single, additive and interaction effects among the environmental and density covariates. Models were ranked using AIC values, considering a ΔAIC of two, to identify the model of best fit [45]. Finally, we extracted the covariate parameter estimates from the model of best fit, which indicated the strength and direction of the relationships of each covariate with survival and recapture.

## Results

### Nestling survival

Model selection revealed the best-fit model included additive fixed effects of Rain12, SOI, hatch date, density and random effects of year and pair ID on survival. Survival was negatively associated with Rain12 during the nestling period (Table 1), meaning that as rainfall increased nestling survival decreased. SOI was positively related to survival, meaning that survival was higher when El Niño cycles were weaker. Hatch date was positively related to survival, thus survival increased with hatch date, indicating that the later hatching occurs, the high survival probability the chick will have. Density was negatively related to survival, meaning that nestling survival decreased as density of juveniles and adults on the island increased.

**Table 1:** Parameter estimates for the best-fit model of nestling survival with standard error.

	Rain12	SOI	Hatch Date	Density
Effect Size	-0.18 ± 0.03	0.12 ± 0.05	0.19 ± 0.04	-0.32 ± 0.08

Treating Rain12 as two variables, corresponding to mean rainfall in the first and second half of the nesting period (RainF6 and RainL6) did not produce a better-supported model compared to treating mean rainfall across the nesting period as a single variable. However, in the model that did include these two rainfall variables, the effect of rainfall during the first six days was significant (-0.11; p = 0.002), while that in the last six days it was not (-0.03; p = 0.459). Survival decreased with rainfall over the first six days, and was virtually constant for the remaining six days. This could be explained by a quadratic effect of rainfall. However, the model including a quadratic effect of rainfall was not statistically supported, possibly because it removes a degree of freedom. The list of models can be found in the S2 Table.

### Juvenile and adult survival

**Fig 1:**
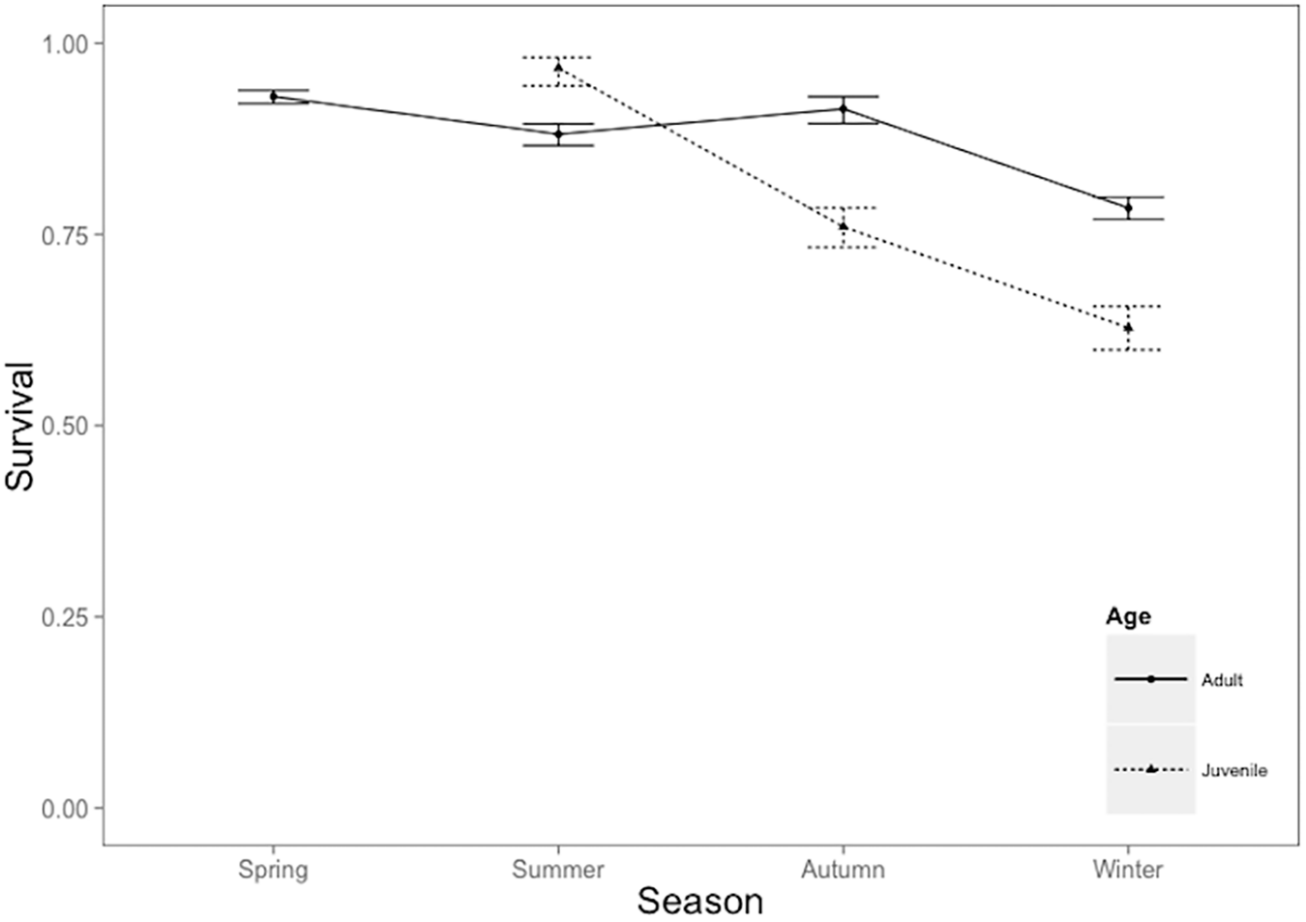
Survival estimates from best-fit model (Season x Age + N + N x Age + SOI + SR + N x SR) for juveniles (dotted lines) and adults (black lines) across seasons.

The survival model of best fit included an interaction between season and age, density and age, additive effects of density, SOI and SR and an interaction between density and SR (S3 Table). Survival varied among seasons and between age classes (Table 2). Juvenile survival was high during summer and decreased during autumn and winter. Adults had a higher survival rate overall, compared to juveniles (0.81 and 0.59, respectively), the highest being 0.93 in spring, 0.88 in summer, 0.91 in autumn, and declining to 0.78 over winter (Fig 1). The overall recapture probability for juveniles was 0.39 and for adults was 0.63.

**Table 2:** Parameter estimates from best-fit model for juveniles and adults survival, with standard error.

	Spring	Summer	Autumn	Winter
Juvenile survival	-	0.96 ± 0.29	0.76 ± 0.07	0.63 ± 0.06
Adult survival	0.93 ± 0.06	0.88 ± 0.06	0.91 ± 0.11	0.78 ± 0.04
Recapture estimates	0.19	0.60	0.42	0.46

**Fig 2:**
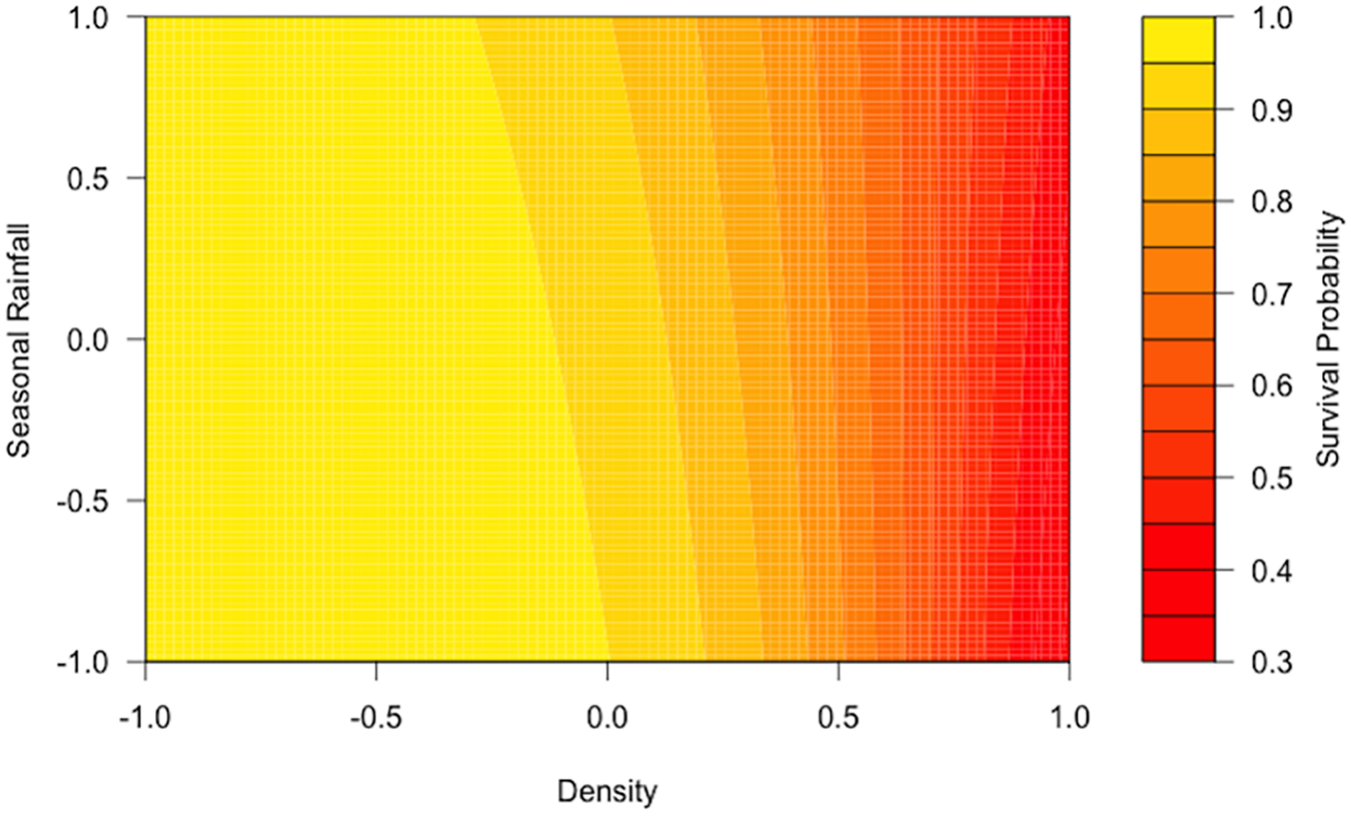
Interaction plot between standardized density and SR in the best-fit model of juvenile and adult survival. The color scale represents survival probabilities, high values in the color scale (yellows) represent high survival probabilities and low values in the color scale (reds) represent low survival probabilities. At lower densities and mean rainfall, survival estimates are higher (light yellow), high density results in lower survival estimates (dark red) and survival decreases slightly as rainfall increases at mid and high densities.

In the best-fit model, density, SOI and SR all showed a negative effect on survival (-3.08, -0.11, -0.38, respectively), while the interaction between density and SR was positive (0.56). Survival was highest at times of low density and low SR (Fig 2).

## Discussion

This study demonstrates that climatic fluctuations and population density are key factors affecting survival in an insular passerine, where the local scale climate explained survival across all age classes the best. For nestlings, the direct impact of rainfall, SOI, hatching date and density were important factors influencing survival. For adults and juveniles, years of higher rainfall were detrimental to survival when density was also high, suggesting a climate-density interaction.

Survival probabilities were high both for juveniles and adults, as expected for subtropical passerines [22,49]. Our results concord with survival estimates obtained by Clegg et al. 2008 [50]from this population in a more recent period (1999–2003), where adults also maintained year-around high survival (0.81) and juvenile survival varied seasonally, with lower survival in winter (~0.40). Our survival estimates on the island are also higher than those previously reported from the mainland (0.61)[51]. The interaction between rainfall and density for adults and juveniles predicts low survival when both rainfall and density are high, but low survival probabilities when density is at its highest, regardless of the amount of rainfall. Similar findings have been described in seabirds, where poor climatic conditions associated to ENSO interact with density-dependence and decrease survival [24]. Our results are with concordance with a density-dependent population, supporting the results of McCallum et al. (2000) of the same population, were juvenile survival was dependent on adult density.

Rainfall can affect survival directly (e.g. thermoregulation, reduced foraging opportunities or insect activity)[52] or indirectly (e.g. lag effect of fruit and/or insect availability)[53,54]. Our results suggest that rainfall was more likely to be influencing survival directly via thermoregulation of individuals, reduced foraging opportunities during events of harsh weather. Nestling survival would most likely be affected via thermoregulation, since in most passerines nestlings are not able to thermoregulate immediately after hatching, but develop it gradually [55]. This would also explain the significant effect of rainfall during the first six days of the nestling period, but not during the last six days, when we tested the rainfall effect as two variables. Moreover, adults spend more time brooding nestlings [52] and less time foraging during rainfall [56]; this implies that parents are spending less time feeding themselves, as well as their offspring, and could be a factor that explains the SR effect on survival of silvereye adults. Juveniles may be experiencing increased starvation from a combination of the effects of reduced foraging time, decreased insect activity and weak foraging skills [57]. Additionally, we found no support for an indirect lagged effect of SR; which could be related to increases in food availability after rainfall. This may be due to the lagged variable not accurately matching with food availability or perhaps food resources were not a constraint during the study period. Direct measures of seasonal food availability would allow us to gain insight into its link with rainfall, but such data were unavailable.

Studies on other islands have assessed the demographic impact of food availability on bird populations. Grant et al. [13,58] showed how heavy rainfall in the Galapagos boosted insect and seed availability, which resulted in drastic increases of fledgling production in two species of finches, altering the size and age class structure of the population. In the Canary Islands, rainfall-mediated food availability influenced the onset of breeding in Stonechats (*Saxicola dacotiae*), but did not significantly alter fledgling success (i.e. nestling survival)[59]. However, these cases show how rainfall-mediated food availability influences aspects of demography in arid environments, where scarce rainfall is a constraint on food availability [60]. In contrast, our study site is located in a subtropical zone where there are periods of rain throughout the year, and food resources, namely insects and figs, are available year-round [42]. On Heron Island, Catterall [42] previously calculated proxies for food availability, in terms of potential food resources across the island, and determined that in areas with higher “fig availability” there was a significantly higher fledging success.

However, the link between rainfall and “fig availability” was not determined. Furthermore, our results did not show evidence of a food-mediated impact on survival through a lagged-rainfall effect. Thus, the specific mechanism by which rainfall and rainfall-mediated food availability impacts nestling survival remains unclear.

In regard to the hatching date, our findings may be in contrast to much of the literature, where nestling or fledgling survival decreases as the breeding season goes on [61–67], albeit most of this literature is from studies that have been conducted in the northern hemisphere. In the southern hemisphere other studies in passerines have reported similar findings, where nestling survival increased slightly as the breeding season progresses [68], attributed to higher likelihood of poor weather early in the breeding season. However, more research is needed to understand the causes and if this pattern is found throughout the southern hemisphere.

High population density was detrimental to nestling survival. Similar findings have been found for birds breeding in the northern hemisphere, where breeding success is density dependent [69–71]. A suggested driver of the density-dependence has been pair competition for food resources. This is one of the likely factors influencing nestling survival at our study site, as it has been previously supported by Catterall et al. 1982 [42], where they found that pairs with territories containing increased access to food resources (measured in number of fig trees per territory) fledged more young. However, they also found that the parents’ age and capacity to find insects were important factors in fledging success. Where older parents and those who fed more insects to their nestlings were able to fledge more young.

In recent years the use of large-scale climate pattern indices to study the effects of climatic variation on population dynamics have often proven to be better predictors of ecological processes than weather measured at a local scale [26,28]. The weak support from our models for a role of SOI may be due to a weak relationship between SOI and rainfall in Australia during the study period [72]. The strength of the relationship between SOI and rainfall in Australia can vary in decadal phases [72]. This may explain why, even though ENSO events influence rainfall patterns, SOI was not able to explain variation in survival as well as locally measured rainfall. Thus, it remains to be tested if during periods when rainfall and SOI are strongly correlated, SOI would be a better predictor of variation in survival. Nonetheless, it is important to include both local and large-scale climate patterns when modeling the effects of climate on population dynamics in order to not overlook aspects of the relationship.

The frequency of El Niño events is predicted to increase [73], which would result in dryer conditions in eastern Australia [74]. According to our results, less rain would be predicted to have a positive impact on survival across all age classes. Moreover, a previous study on Heron Island showed rainfall to be important in the onset of nesting in silvereyes [75], presumably linked to insect and fig availability. Additionally, postponed nesting triggered by less rain could also contribute to higher survival, as our nestling model showed higher survival for nestlings hatched later in the breeding season. However, the long-term effect of lower rainfall on insect and fig abundance on the island is difficult to predict, but would most likely be negative. The long-term dynamics of the population will depend on the outcome of the positive influences of the predicted lower rainfall on survival and if the effect of lower rainfall on food availability becomes a constraint on survival across age classes.

Our results contribute to our understanding of the population dynamics of subtropical insular passerines, and the links between stochastic environmental and density-dependent effects. In this case rainfall was a key factor, interacting with density, while large-scale climate pattern indexes were unable to explain individual variation in survival at such a local scale. Future studies should incorporate measurements of food availability in the models, to differentiate between direct and indirect effects of climatic variation on survival probabilities.

## Supporting information

S1 Table (A) First stage of the transition matrix used in E-SURGE for juvenile and adult models.–(B) Second stage of transition matrix used in E-SURGE for juvenile and adult models.–(C) Event matrix used in E-SURGE for juvenile and adult models.
(DOCX)

S2 Table Model selection results for GLMMs of nestling survival probabilities and additive fixed and random effects. (DOCX)

S3 Table Model selection results for survival analysis of juvenile and adult silvereyes. (DOCX)
