# Supplementary material for: The influence of climatic variation and density on the survival of an insular passerine Zosterops lateralis
Source: PLoS One. 2017 Apr 28;12(4):e0176360. doi: 10.1371/journal.pone.0176360 (PMC5409077; doi:10.1371/journal.pone.0176360)
Supplement: S1 Table — (A) First stage of the transition matrix used in E-SURGE for juvenile and adult models.–(B) Second stage of transition matrix used in E-SURGE for juvenile and adult models.–(C) Event matrix used in E-SURGE for juvenile and adult models. (DOCX) [file pone.0176360.s001.docx]

**S1A Table.** First stage of the transition matrix used in E-SURGE for juvenile and adult models.

|  | Trap Aware | Trap Unaware | Dead |
| --- | --- | --- | --- |
| Trap Aware | y | - | * |
| Trap Unaware | - | y | * |
| Dead | - | - | * |

**S1B Table.** Second stage of transition matrix used in E-SURGE for juvenile and adult models.

|  | Trap Aware | Trap Unaware | Dead |
| --- | --- | --- | --- |
| Trap Aware | y | * | - |
| Trap Unaware | y | * | - |
| Dead | - | - | * |

**S1C Table.** Event matrix used in E-SURGE for juvenile and adult models.

|  | ‘0’ | ‘1’ |
| --- | --- | --- |
| Trap Aware | - | * |
| Trap Unaware | * | - |
| Dead | * | - |

For interpretation of these tables please refer to Pradel and Sanz-Aguilar 2012.
