# Supplementary material for: The influence of climatic variation and density on the survival of an insular passerine Zosterops lateralis
Source: PLoS One. 2017 Apr 28;12(4):e0176360. doi: 10.1371/journal.pone.0176360 (PMC5409077; doi:10.1371/journal.pone.0176360)
Supplement: S2 Table — (DOCX) [file pone.0176360.s002.docx]

**S2 Table.** Model selection results for GLMMs of nestling survival probabilities and additive fixed and random effects.

| Model Structure | AIC | ΔAIC | K |
| --- | --- | --- | --- |
| **Rain12 + SOI + Hatch Date + Density** | **6379.5** | **0** | **7** |
| Rain12 + Rain12^2^ + SOI + SOI^2^ + Hatch Date + Density | 6381.3 | 1.73 | 9 |
| Rain12 + Hatch Date + Density | 6382.8 | 3.24 | 6 |
| Rain12 + SOI + Hatch Date | 6392.2 | 12.59 | 6 |
| RainF6 + RainF6^2^ + RainL6 + RainL6^2^+ SOI + SOI^2^ + Hatch Date + Density | 6392.4 | 12.94 | 11 |
| RainF6 + RainF6^2^ Hatch Date + Density | 6393.4 | 13.83 | 7 |
| RainF6 + RainF6^2^ + RainL6 + RainL6^2^ Hatch Date + Density | 6394.5 | 15.01 | 9 |
| RainF6 + RainL6 + Hatch Date + Density | 6398.4 | 18.78 | 8 |

For each model, the AIC, the difference in AIC from the best model (ΔAIC) and the number of parameters (K) is provided. The model of best fit is highlighted in bold. Description of fixed effect parameters can be found in the methods. All models include random effects of Year and Pair ID.
