# Supplementary material for: The influence of climatic variation and density on the survival of an insular passerine Zosterops lateralis
Source: PLoS One. 2017 Apr 28;12(4):e0176360. doi: 10.1371/journal.pone.0176360 (PMC5409077; doi:10.1371/journal.pone.0176360)
Supplement: S3 Table — (DOCX) [file pone.0176360.s003.docx]

**S3 Table.** Model selection results for survival analysis of juvenile and adult silvereyes.

| Model Structure | AIC | ΔAIC | K |
| --- | --- | --- | --- |
| **Season x Age + N + N x Age + SOI + SR + N x SR** | **36054.43** | **0** | **20** |
| Season x Age + N + N x Age + SOI + SR + N x SOI | 36068.95 | 14.52 | 20 |
| Season x Age + N + N x Age + SOI + SR | 36076.09 | 21.66 | 19 |
| Season x Age + N + SOI + SR + N x SR | 36077.20 | 22.77 | 18 |
| Season x Age + N + N x Age + SR + N x SR | 36082.43 | 28.00 | 19 |
| Season x Age + N + N x Age + SOI | 36088.28 | 33.85 | 18 |
| Season x Age +N + SOI + SR | 36109.87 | 55.44 | 17 |
| Season x Age + N + lagSOI | 36109.99 | 55.56 | 16 |
| Season x Age + N + N x Age | 36114.09 | 59.66 | 17 |
| Season x Age + N + SR + NxSR | 36123.72 | 69.29 | 17 |
| Season x Age + N + SOI +NxSOI | 36123.90 | 69.47 | 17 |
| Season x Age + N + lagSR | 36124.15 | 69.72 | 16 |
| Season x Age + N + lagSOI + NxlagSOI | 36128.72 | 74.29 | 17 |
| Season x Age + N + SR | 36144.38 | 89.95 | 16 |
| Season x Age + N + SOI + SR + NxSOI | 36147.24 | 92.81 | 18 |
| Season x Age + SR + SR^2 | 36149.05 | 94.62 | 16 |
| Season x Age + N + lagSR NxlagSR | 36172.76 | 118.33 | 17 |
| Season x Age + N + SOI | 36174.88 | 120.45 | 16 |
| Season x Age + lagSOI | 36180.74 | 126.31 | 15 |
| Season x Age + N + SOI + SR+ lagSR | 36189.22 | 134.79 | 18 |
| Season x Age | 36212.09 | 157.66 | 14 |
| Season x Age + SOI | 36274.81 | 220.38 | 15 |
| Season x Age + SR | 36291.79 | 237.36 | 15 |
| Season x Age + N | 36592.32 | 537.89 | 15 |
| Season x Age + lagSR | 36857.51 | 803.08 | 15 |
| Season x Age + SOI + SOI^2 | 37125.35 | 1070.92 | 18 |
| Intercept | 39325.16 | 3270.73 | 3 |

For each model, the AIC, the difference in AIC from the best model (ΔAIC) and the number of parameters (K) is provided. The model of best fit is highlighted in bold. “Season” denotes a seasonal effect, “Age” an effect of juvenile versus adult age classes, “N” a density effect, “SOI” a three month mean Southern Oscillation Index (SOI) effect, “lagSOI” a one-season lag SOI effect, “SR” a three month mean seasonal rainfall effect, and “lagSR” a one-season lag seasonal rainfall effect.
